# How spontaneous pneumothorax is managed in emergency departments: a French multicentre descriptive study

**DOI:** 10.1186/s12873-018-0213-2

**Published:** 2019-01-11

**Authors:** S. Kepka, J. C. Dalphin, J. B. Pretalli, A. L. Parmentier, D. Lauque, G. Trebes, EXPPI study group, B. Mazet, J. Schmidt, D. Honnart, G. Trebbes, J. Y. Lardeur, D. Lauque, J. B. Braun, A. E. Dubart, ED, G. Duncan, N. Bronet, B. Goulesque, A. Delpechin, T. El Cadi, F. Mauny, T. Desmettre

**Affiliations:** 1 Emergency department, CHRU of Strasbourg – Nouvel hôpital civil, 1 place de l’hopital, 67091 Strasbourg, France; 2 UMR 6249 Chronoenvironnement/University of Franche Comté, La Bouloie - UFR Sciences et Techniques, 16 route de Gray, 25030 Besançon cedex, France; 3 Department of respiratory diseases, CHRU of Besançon, 1 boulevard Fleming, 25030 Besançon, France; 4 MSC Clinical Methodology Center, CHRU of Besançon, 2 place Saint Jacques, 25030 Besançon, France; 5 Emergency department, CHRU of Besançon, 1 boulevard Fleming, 25030 Besançon, France; 6 Emergency department, CHRU of Toulouse, Place du Docteur Baylac – TSA 40031, 31059 cedex 9 Toulouse, France; 7 Emergency department, La Tronche - CHRU of Grenoble, Boulevard de la Chantourne,, 38700, cedex 9 La Tronche, France

**Keywords:** Spontaneous pneumothorax, Outpatient management, Observation, Aspiration, Thoracic drainage, Emergency department

## Abstract

**Background:**

Management of spontaneous pneumothorax (SP) is still subject to debate. Although encouraging results of recent studies about outpatient management with chest drains fitted with a one-way valve, no data exist concerning application of this strategy in real life conditions. We assessed how SP are managed in Emergency departments (EDs), in particular the role of outpatient management, the types of interventions and the specialty of the physicians who perform these interventions.

**Methods:**

From June 2009 to May 2013, all cases of spontaneous primary (PSP) and spontaneous secondary pneumothorax (SSP) from EDs of 14 hospitals in France were retrospectively included. First line treatment (observation, aspiration, thoracic drainage or surgery), type of management (admitted, discharged to home directly from the ED, outpatient management) and the specialty of the physicians were collected from the medical files of the ED.

**Results:**

Among 1868 SP included, an outpatient management strategy was chosen in 179 PSP (10%) and 38 SSP (2%), mostly when no intervention was performed. Only 25 PSP (1%) were treated by aspiration and discharged to home after ED admission. Observation was the chosen strategy for 985 patients (53%). In 883 patients with an intervention (47%), it was performed by emergency physicians in 71% of cases and thoracic drainage was the most frequent choice (670 patients, 76%).

**Conclusions:**

Our study showed the low level of implementation of outpatient management for PS in France. Despite encouraging results of studies concerning outpatient management, chest tube drainage and hospitalization remain preponderant in the treatment of SP.

**Electronic supplementary material:**

The online version of this article (10.1186/s12873-018-0213-2) contains supplementary material, which is available to authorized users.

## Background

*S*ignificant questions remain regarding the optimal initial approach in the management of spontaneous pneumothorax (SP) [1, 2], with no superiority of thoracic drainage and aspiration [3, 4]. In the absence of international consensus, the choice of first line treatment and the possibility of outpatient management are left at the discretion of the practitioners taking care of these patients.

Ambulatory care is a way to reduce both the duration of hospitalization and crowding in Emergency Departments (EDs). SP is generally considered as a benign pathology occurring in young people, which implies a possibility of ambulatory management. Pilot studies have proven the feasibility of ambulatory management for SP [5–9]. However, real-life implementation of outpatient management for patients with SP is actually unknown.

Healthcare pathways for SP have been elucidated in a recent study by Bobbio et al. in a large population of more than 40,000 patients. The hospitalization rate in medicine units was 73%, versus 27% in surgery [10]. However, this report, based on data from the French national hospitalization database, did not inform about the effective first line treatment in the ED. Another multicenter study conducted in EDs in Australia did not provide any data concerning the type of equipment, the speciality of the physicians managing the patients, or the departments of hospitalization [11]. Currently, there exists a gap in knowledge concerning real-life conditions of SP management in the ED, in particular the role of ambulatory management and the type of interventions performed.

In this context, we report here the real-life management in the ED of SP including both primary and secondary spontaneous pneumothorax. Our aims were to describe how SP is managed in the ED, particularly the proportion of outpatient management.

## Methods

### Study design

A retrospective observational study was conducted between 1 June 2009 and 31 May 2013 in 14 emergency departments (ED) of hospitals geographically distributed across the whole of France and members of the EXPRED network. Participating centers also had to use an electronic patient file in the ED to be eligible. Based on these criteria, seven French university teaching hospitals and seven non-academic hospitals were involved. Hospitals varied in terms of annual ED admissions, ranging from 22,159 to 100,619, and in total represented approximately 711,200 visits per year. The list of investigators from each participating ED is reported in Additional file 1.

### Study setting and population

Inclusion criteria for SP cases were: age over 18 years and a main diagnosis of primary spontaneous pneumothorax (PSP) or secondary spontaneous pneumothorax (SSP). SSP was defined as pneumothorax occurring with the knowledege of at least one pre-existing lung disease i.e. Chronic Obstructive Pulmonary Disease (COPD), emphysema, or chronic respiratory failure. Non-inclusion criteria were patients with traumatic pneumothorax, defined as a history of thoracic trauma. For this type of study, formal written informed consent was not required. A non-opposition procedure was used and no patient had expressed opposition to the use of his medical data for research purposes.

### Endpoints

The primary endpoint was the description of first line treatment in the ED for both PSP and SSP and whether patients were managed in-hospital or as outpatients.

Treatments were recorded, and classified as follows:

- Observation, for patients in whom no interventional procedure to restore an air-free pleural space was performed.
- Aspiration, for patients in whom simple needle aspiration or a pleural drainage catheter was used, with withdrawal of the equipment at the end of the aspiration procedure [12].
- Thoracic drainage, for patients in whom the drainage equipment was left in place, regardless of whether it was connected to a collection device.
- Any type of surgery was classified as surgery.

Outpatient management was defined as patients discharged to home directly from the ED and not admitted to a hospital ward.

Secondary endpoints were to describe the type of equipment used for the procedure in case of intervention, as well as the specialty of the physicians who performed these procedures. Equipment types included: thoracocentesis needle, pigtail catheter (Cook, Bloomington, USA), Pleurocath® (Prodimed, Neuilly en Thelle, France), pleural puncture device for aspiration, central venous catheters. Thoracic drainage could be performed with a standard 12 to 24 Fr chest tube, a pigtail catheter, or a Pleurocath® device. The speciality of the physician performing the treatment in the ED was recorded as one of the following: emergency physician, respiratory physician, surgeon, intensive care physician. Finally, in case of admission, the type of ward was recorded (short stay medical unit, department of respiratory diseases, intensive care unit).

### Measurements

Patients were identified in the ED admissions databases of the participating centers from the medical informatics programs (software programmes: *Resurgences® (Berger Levrault, Boulogne-Billancourt, France), Urqual® (Maincare Solutions, Cestas, France), TralcareClinicom® (Intersystems, Cambridge, USA), Cristalnet® (Alma,Grenoble, France)*. All patients with a primary diagnosis of pneumothorax (International Classification of Diseases (ICD) 10th revision code = J93) during the study period were retrospectively identified by medical informatics queries. Among these patients, a diagnosis of PSP (ICD code J93.11) or SSP (ICD code J93.12) was selected by research assistants in the medical files of the EDs. Furthermore, for the description of treatment in EDs, patients that were included a randomized trial that was ongoing at the time, were not retained for the analysis of the treatment to avoid bias.

Age, gender, smoking habits (current smokers, ever smokers or non-smokers), self-reported use of cannabis, documented history of pneumothorax in the patient’s medical file were recorded.

Clinical tolerance of SP was evaluated by analysis of clinical data, namely respiratory rate, blood pressure, heart rate, and SpO2 in ambient air were also collected. Numerical pain score at admission was recorded (on a numerical rating scale from 0 to 10). Presence of dyspnea was also investigated by checking the patient’s medical file. Signs of gravity were considered as the presence of chest pain, dyspnea or hypoxemia with SpO2 ≤ 90%.

### Analysis

Quantitative variables are described as mean ± standard deviation, and categorical variables as number and percentage. The Student t, chi-square or Fisher’s exact tests were used as appropriate. Statistical significiance was set at *p* < 0.05. Analyses were performed using R Version R 3.2.0 (R Foundation for Statistical Computing, Vienna, Austria).

## Results

Among 3088 cases of pneumothorax identified, 1098 (35.6%) were traumatic and were thus excluded from further analysis. A total of 1990 SP were studied: 1632 (82%) PSP and 358 (18%) SSP. The details of the 1990 SP are presented in Table 1. Clinical data and vital parameters of SP are presented in Table 2. When considering hypoxemia, patients with SP had a good tolerance (SpO2 ≤ 90% in only 3.5%). SpO2 was significantly higher for PSP than for SSP (*p* < 0.0001). Furthermore, patients with PSP often declared dyspnea but had less chest pain than SSP (*p* < 0.0001). For analysis of treatment, only 1868 patients were studied: 122 patients could not be analyzed because of their participation in a randomized trial over this period, concerning the therapeutic strategy (EXPRED study).

**Table 1:** Characteristics of patients

Characteristics		All SP	PSP	SSP	*p*
		(*n* = 1990)	(*n* = 1632)	(*n* = 358)	
Sex					0.65
	Female, n (%)	399 (21.8%)	351 (21.6%)	48 (23%)	
	Male, n (%)	1435 (78.2%)	1274 (78.4%)	161 (77%)	
Age (years), mean (SD)		33.2 (15.6%)	30.4 (12.6%)	54.4 (19.9%)	< 0.0001
Smoking history					< 0.0001
	Current smoker, n (%)	885 (67.2%)	787 (68.3%)	98 (60.12%)	
	Ex-smoker, n (%)	143 (10.9%)	89 (7.7%)	54 (33.13%)	
	Never smoker, n (%)	288 (21.9%)	277 (24%)	11 (6.75%)	
Cannabis, n (%)		81 (4.4%)	77 (4.7%)	4 (1.9%)	0.07
Previous history of PS, n (%)		708 (38.5%)	619 (37.9%)	89 (43%)	0.15

*SP* spontaneous pneumothorax

*PSP* primary spontaneous pneumothorax

*SSP* secondary spontaneous pneumothorax

Data are presented as mean and standard deviation (SD)

**Table 2:** Clinical tolerance and vital parameters of spontaneous pneumothorax

Clinical data and vital parameters		All SP	PSP	SSP	
		(*n* = 1990)	(*n* = 1632)	(*n* = 358)	
Chest pain					< 0.001
	Yes, n (%)	1615 (90.7%)	1470 (93.1%)	145 (72.1%)	
	No, n (%)	165 (9.3%)	109 (6.9%)	56 (27.9%)	
Dyspnea					< 0.001
	Yes, n (%)	787 (45.3%)	644 (41.8%)	143 (72.6%)	
	No, n (%)	949 (54.7%)	895 (58.2%)	54 (27.4%)	
Pain score, mean (SD)		4.18 (2.3%)	4.2 (2.7%)	3.5 (3.1%)	0.01
SpO2 (%)					< 10–3
	< 90%, n (%)	37 (2.5%)	19 (1.4%)	18 (12.5%)	
	≥90%, n (%)	1464 (97.5%)	1338 (98.6%)	126 (87.5%)	
Respiratory rate (per minute)					0.005
	< 10 or > 20, n (%)	307 (44%)	256 (42%)	51 (58%)	
	≥10 or ≤ 20, n (%)	391 (56%)	354 (58%)	37 (42%)	
Heart rate (per minute)					0.002
	< 40 or > 120, n (%)	68 (4.1%)	52 (3.5%)	16 (8.6%)	
	≥40 or ≤ 120, n (%)	1580 (95.9%)	1411 (96.5%)	169 (91.4%)	
Systolic blood pressure (mmHg)					0.61
	< 90, n (%)	10 (0.6%)	10 (0.7%)	0	
	≥90, n (%)	1644 (99.4%)	1455 (99.3%)	189 (100%)	

Pain score on a numerical range from 0 to 10

**Fig. 1:**
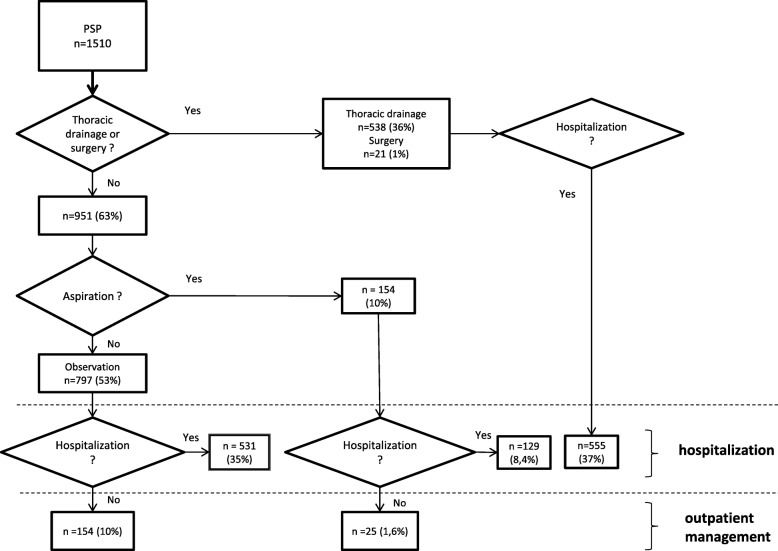
Management of Primary Spontaneous Pneumothorax in ED

**Fig. 2:**
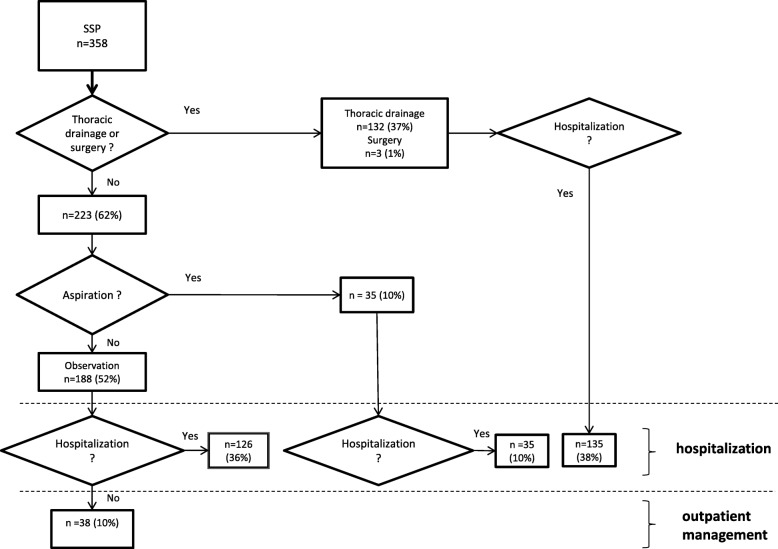
Management of Secondary Spontaneous Pneumothorax in ED

Figures 1 and 2 present respectively the first line treatments for PSP and SSP (*n* = 1868), as well as the hospitalization and discharge rates. In this series, among the 1868 PS, 883 (47%) required an intervention and 985 observations (53%).

Among the 985 observations, 192 (20%) were outpatient management and 657 (67%) were managed in hospital.

Concerning patients with intervention (*n* = 883), only 25 PSP (3%) with aspiration in the ED had outpatient management.

Thus, outpatient management was the retained strategy for 179 PSP (10%) and for 38 SSP (2%). For PSP, observation concerned 154 patients (8%) and 25 patients (1%) were treated by aspiration. For SSP, outpatient management concerned only patients when no intervention was required. No patient in our series had outpatient management with a pigtail catheter. In case of intervention, thoracic drainage was the technique most commonly chosen by the practitioners. Interventions were performed by an emergency practitioner in 478 patients (71%).

Table 3 presents the type of equipment used for the procedures and the specialty area of the physicians who performed the interventions. Among the 1868 SP, 217 patients were discharge to home after ED admission (12%) and 1508 patients were admitted, distributed as follows: 639 (34%) to a surgery unit, 638 to the department of respiratory disease (34%), 164 to a short stay unit (9%) and 67 to the intensive care unit (4%). In this retrospective study, data concerning outcomes are missing for 143 patients.

**Table 3:** Type of equipment used and speciality of physicians for aspiration and thoracic drainage

Type of treatment	Category		All SP*	PSP*	SSP	*p*
			*n* = 1868	*n* = 1510	*n* = 358	
Thoracic drainage (*n* = 670)						
	Equipment					0.07
		Chest tube, n (%)	568 (91%)	462 (92%)	106 (86.9%)	
		Pleurocath, n (%)	52 (8.3%)	38 (7.6%)	14 (11.5%)	
		Pigtail catheter, n (%)	1 (0.2%)	0	1 (0.8%)	
		Other, n (%)	3 (0.5%)	2 (0.4%)	1 (0.8%)	
	Speciality of physicians					0.3
		Emergency physician, n (%)	363 (68.8%)	285 (68.7%)	78 (69%)	
		Respiratory medicine, n (%)	56 (10.6%)	40 (9.6%)	16 (14.2%)	
		Surgeon, n (%)	46 (8.7%)	36 (8.7%)	10 (8.8%)	
		Intensive care physician, n (%)	63 (11.9%)	54 (13%)	9 (8%)	
Aspiration (*n* = 189)						
	Equipment					0.58
		Needle thoracocentesis, n (%)	58 (80.6%)	50 (80.6%)	8 (80%)	
		Catheters, n (%)	7 (9.7%)	6 (9.7%)	1 (20%)	
		Other, n (%)	4 (5.6%)	4 (6.5%)	0	
		Pleurocath, n (%)	3 (4.2%)	2 (3.2%)	0	
	Speciality of physicians					0.12
		Emergency physician, n (%)	115 (79.9%)	94 (81%)	21 (75%)	
		Respiratory medicine, n (%)	17 (11.8%)	11 (9.5%)	6 (21.4%)	
		Surgeon, n (%)	9 (6.3%)	9 (7.8%)	0	
		Intensive care physician, n (%)	3 (2.1%)	2 (1.7%)	0	

*SP* spontaneous pneumothorax

*PSP* primary spontaneous pneumothorax

*SSP* secondary spontaneous pneumothorax

*122 patients excluded from analysis because randomised in the EXPRED study

## Discussion

In this description of ED treatment strategies in 1868 SP, outpatient management concerned only 1% of SP in EDs in patients with an intervention, and there was no outpatient management with pigtail catheters, even though the majority of SP were well tolerated. Observation was the first line approach in half of the cases. When an intervention was performed, thoracic drainage was chosen in 76% of cases and performed mainly by emergency physicians.

To the best of our knowledge, this represents the largest series to date detailing first line treatment of SP in the ED, and management strategies in real life conditions.

Outpatient management is a useful way to reduce crowding in the ED, with considerable cost savings. In our study, only 25 patients (1, 3%) who underwent intervention had outpatient management, and all these patients had a PSP treated by aspiration. Our results thus confirm those of a recent study including 72 patients, with an ambulatory device attached to a chest drain for 5% of them [12]. Nevertheless, ambulatory management appears to be a promising alternative. In 2012, Lai et al. included 55 patients with a first episode of PSP treated by small-bore (8 Fr) chest drains fitted with a one-way Heimlich valve, with a success rate of 65.5% [13]. This was confirmed in 2014 in a study by Voisin et al., with a succes rate of 78% in patients managed exclusively as outpatients, reaching 84% in patients with a first primary spontaneous pneumothorax (PSP) without major complications [9]. Comparable rates of success were also reported [14]. A systematic review by Brims et al. retained 18 studies [15]. The overall quality was moderate to poor with high risk of bias. Only one study compared aspiration and ambulatory devices with a Heimlich valve [8]. The success rate was similar in the 2 groups. Fifty-two percent and 28% of patients with aspiration and chest tube were admitted from the ED to the inpatient ward; 9 and 12% of patients who had aspiration and chest tube respectively were admitted from the review clinic. However, this study included only 48 patients and the criteria for success were different in the 2 groups, which could explain the difference concerning hospitalization rates. Nonetheless, high quality studies of clinical efficacy are warranted to compare the use of Heimlich valves with intercostal catheters, versus standard tube thoracostomy, versus needle aspiration [4, 16]. Although this former strategy appears safe, complications have been described and must be taken into account [17].

Furthermore, questions have arisen concerning the application of outpatient management [18], in particular which physicians perform the intervention. In the study by Voisin et al., all pigtail catheters were placed by respiratory physicians [9], but emergency physicians now place the majority of tubes, as confirmed in our study and others [5]. It implies that the choice of the safest and most cost effective strategy is a real challenge for emergency physicians. In addition, the main barrier to the widespread implementation of this strategy concerns the follow-up after ED discharge. In the report by Trueger et al. from the Global Emergency Medicine Journal Club, no commentators reported having an institutional protocol for arranging outpatient follow-up with pulmonologists, as in the study algorithm of Voisin et al. [9], and they report that it appears difficult to establish a program for outpatient monitoring by pulmonologists or cardiothoracic surgeons [18]. Development of postemergency consultations is essential, and this approach has shown good results for follow-up of patients in other pathologies [19].

In our study, abstention was the first line treatment for half of all patients, with outpatient management for only 192 patients (19%). No consensus exists concerning the follow-up and monitoring, whether in ambulatory care or in-hospital [1, 2]. One possibility for monitoring of these patients could be admission to short-stay medical units. However, in our study, only 9% were admitted to this type of ward. These units are a good alternative in the context of ED overcrowding. In 2002, the UK government introduced a target of four hours from arrival at the ED to discharge or admission, for its emergency departments [20], but this system had some limitations [21]. The American College of Emergency Physicians recommends short-stay units as a possible solution to alleviate this problem, although the evidence for their utility remains controversial. Indeed, a systematic review published in 2015 concluded that there is insufficient evidence to make conclusions regarding the effectiveness and safety of short-stay medical units, compared with inpatient care [22].

Finally, our study confirms the high rate of use of thoracic drainage as first line treatment in case of intervention. These results are in line with those of two studies conducted in the Republic of Ireland and in the UK, which included respectively 20 and 57 patients [23, 24]. They showed that compliance with the recommandations of the British Thoracic Society was poor, and aspiration was performed respectively in 5 and 16% of patients. Likewise, Brown et al. reported that thoracic drainage was chosen for more than 80% of PSP and SSP with an intervention [11]. As in our study, SP was well tolerated and hypoxemia occurred mostly in SSP [11].

Chest tubes were more often used than less invasive equipment such as pleurocath or pigtail catheters, even though small bore chest drains are recommended [1, 25, 26]. Some barriers to the use of less invasive strategies include the lack of reliable specific equipment, low awareness of techniques by physicians who are more accustomed to conventional drainage, or the absence of therapeutic consensus [27]. Currently, the “technical” gap between aspiration and drainage is closing, with various devices that can either be left in place or removed rapidly once the pleural space has been rendered air-free [28]. There is considerable variation in the definitions used between studies, with the same terms sometimes having different meanings. Baumann et al. proposed a definition of pleural drainage catheters that included needle thoracocentesis and small bore chest drains [29]. In any case, the distinction between aspiration and drainage is progressively giving way to a choice between management strategies, namely ambulatory management or conventional management with drainage. Pleural drainage by catheter, with the equipment either left in place or removed after the intervention offers the advantage of allowing ambulatory management. Another type of material is currently being evaluated in the ongoing RAMPP study (Randomised Ambulatory Management of Primary Pneumothorax, UK clinical trial registered under the number ISRCTN79151659), and should facilitate procedures through the use of a single device (drain and one-way valve). The use of a small intercostal catheter offers an option for outpatient management, and is also relevant thanks to its simplicity of use for emergency physicians.

The main limitation of our study lies in its retrospective design, with the potential biases that this entails. In this regard, we did not record data concerning the size of the pneumothorax or one year recurrence rates, as these points have already been largely reported in the literature [11, 12] and were not the main objective of this study. Although data regarding the size of pneumothorax were not available in this retrospective study, the individual treatment strategy used for each patient was in line with current guidelines and it is unlikely that our study population differs substantially from the general population of patients admitted for this disease. Furthermore, the size of pneumothorax is not consensual according to recommendations of the ACCP and BTS, as previously shown in several studies [3, 4, 31].

Despite the lack of data about the size of pneumothorax, we found that SP were well tolerated and could probably be managed with outpatient management. According to the BTS recommendations [1], the presence of breathlessness influences the management strategy and indicates the need for active intervention as well as supportive treatment. So, selected asymptomatic patients with a large PSP may be managed by observation alone. Then, clinical tolerance and persistence of symptoms such as dyspnea or chest pain is also important in deciding whether a patient can be discharged after the ED as purposed by an ongoing multicentre, prospective, randomised, controlled, open label parallel group, non-inferiority study study by Brown et al. comparing conservative versus invasive treatment of PSP [30]. The hypothesis is that resolution of large PSP will be similar after 8 weeks with either therapeutic regimen. Thus, the size of the pneumothorax does not appear to a criterion in determining the treatment strategy in their study, whereas persistance of significant symptoms such as dyspnea or chest pain, and physiological instability developing during the observation period are retained to decide between discharge from the ED, conservative or invasive strategies.

Despite the multicenter design, we cannot rule out the possibility of selection bias. Indeed, the participating centres were selected among departments involved in a national network (EXPRED study) [31]. However, different type of hospitals, both university teaching hospitals and non-academic (general) hospitals participated. Furthermore, the population of our study had similar characteristics in terms of age, sex ratio, proportion of PSP and SSP as reported in studies by Bobbio and Brown [11, 12]. Another limitation is the fact that we did not record data regarding the availability of different specialists in real time in the participating centres. Thus, our findings, particularly regarding the specialty of origin of the physicians performing the interventions, cannot be put in perspective with the availability of each specialty. Voisin et al. reported this type of data, notably specifying that a pulmonologist could be contacted if necessary day and night [9]. These data would have been interesting and may have been influential in the choice and availability of ambulatory management.

## Conclusions

Despite several limitations, this multicentre study underlines the low level of implementation of outpatient management of PS in France, even though most patients with SP admitted to the EDs had no signs of gravity. Our study confirmed the persistently predominant role of thoracic drainage in the management of SP. Approximately half the patients in our study had no intervention in the ED. A majority of patients were admitted to a hospital unit, whereas many would have been eligible for outpatient management. Considering the encouraging results of recent studies about ambulatory management of PS, we can expect in the future that the proportion of patients with outpatient management will increase. For PS with good tolerance, observation could be proposed as outpatient management in a substantial proportion of cases, in particular for PSP. Outpatient management with a pigtail-catheter with a one-way valve appears a safe strategy but further comparative studies are necessary before implementation of this strategy in real life conditions. The development of specific healthcare pathways in collaboration between emergency physicians, pulmonologists and surgeons is key to wider use of ambulatory management in patients with spontaneous pneumothorax.

## Additional file

Additional file 1: EXPRED Study group. (DOCX 77 kb)

## Abbreviations

ED: Emergency department
PSP: Primary spontaneous pneumothorax
SP: Spontaneous pneumothorax
SSP: Secondary spontaneous pneumothorax
