# Supplementary material for: How spontaneous pneumothorax is managed in emergency departments: a French multicentre descriptive study
Source: BMC Emerg Med. 2019 Jan 11;19:4. doi: 10.1186/s12873-018-0213-2 (PMC6329130; doi:10.1186/s12873-018-0213-2)
Supplement: Supplementary file 1 — EXPRED Study group. (DOCX 77 kb) [file 12873_2018_213_MOESM1_ESM.docx]

B. Mazet, ED, University Hospital of Angers, 49000, France. [BeMazet@chu-angers.fr](mailto:BeMazet@chu-angers.fr)

T. Desmettre, ED, University Hospital of Besançon, 25030, France. [tdesmettre@chu-besancon.fr](mailto:tdesmettre@chu-besancon.fr)

J. Schmidt, ED, University Hospital of Clermont Ferrand, 63100, France. [jschmidt@chu-clermontferrand.fr](mailto:jschmidt@chu-clermontferrand.fr)

D. Honnart, ED, University Hospital of Dijon, 21000, France. [didier.honnart@chu-dijon.fr](mailto:didier.honnart@chu-dijon.fr)

G. Trebbes, ED, University Hospital of Grenoble, 38700, France. gtrebbes@chu-grenoble.fr

J.Y. Lardeur, ED, University Hospital of Poitiers, 68021, France. j.y.lardeur@chu-poitiers.fr

D. Lauque, ED, University Hospital of Toulouse, 31400, France. [lauque.d@chu-toulouse.fr](mailto:lauque.d@chu-toulouse.fr)

J.B. Braun, ED, Hospital of Belfort, 90000, France. [jbbraun@chbm.fr](mailto:jbbraun@chbm.fr)

A.E. Dubart, ED, Hospital of Bethune, 62660, France. [aedubart@sfr.fr](mailto:aedubart@sfr.fr)

G. Duncan, ED, Hospital of Boulogne/Mer, 62200, France. g.ducan@ch-boulogne.fr

N. Bronet, ED, Hospital of Lomme, 59462, France. bronet.nathalie@ghicl.net

B. Goulesque, ED, Hospital of Mulhouse, 68100, France. [goulesqueb@ch-mulhouse.fr](mailto:goulesqueb@ch-mulhouse.fr)

A. Delpechin, ED, Hospital of Roubaix, 59100, France. [Antoine.depelchin@ch-roubaix.fr](mailto:Antoine.depelchin@ch-roubaix.fr)

T. El Cadi, ED, Hospital of Vesoul, 70000, France. [t.elcadi@chi70.fr](mailto:t.elcadi@chi70.fr)
